# Molecular characterization of *Histomonas meleagridis* exoproteome with emphasis on protease secretion and parasite-bacteria interaction

**DOI:** 10.1371/journal.pone.0212429

**Published:** 2019-02-26

**Authors:** Rounik Mazumdar, Katharina Nöbauer, Karin Hummel, Michael Hess, Ivana Bilic

**Affiliations:** 1 Clinic for Poultry and Fish Medicine, Department for Farm Animals and Veterinary Public Health, University of Veterinary Medicine Vienna, Vienna, Austria; 2 VetCORE, Facility for Research, University of Veterinary Medicine Vienna, Vienna, Austria; 3 Christian Doppler Laboratory for Innovative Poultry Vaccines (IPOV), University of Veterinary Medicine Vienna, Vienna, Austria; Hospital for Sick Children, CANADA

## Abstract

The exoproteome of parasitic protists constitutes extracellular proteins that play a fundamental role in host-parasite interactions. Lytic factors, especially secreted proteases, are capable of modulating tissue invasion, thereby aggravating host susceptibility. Despite the important role of exoproteins during infection, the exoproteomic data on *Histomonas meleagridis* are non-existent. The present study employed traditional 1D-in-gel-zymography (1D-IGZ) and micro-LC-ESI-MS/MS (shotgun proteomics), to investigate *H*. *meleagridis* exoproteomes, obtained from a clonal virulent and an attenuated strain. Both strains were maintained as mono-eukaryotic monoxenic cultures with *Escherichia coli*. We demonstrated active *in vitro* secretion kinetics of proteases by both parasite strains, with a widespread proteolytic activity ranging from 17 kDa to 120 kDa. Based on protease inhibitor susceptibility assay, the majority of proteases present in both exoproteomes belonged to the family of cysteine proteases and showed stronger activity in the exoproteome of a virulent *H*. *meleagridis*. Shotgun proteomics, aided by customized database search, identified 176 proteins including actin, potential moonlighting glycolytic enzymes, lytic molecules such as pore-forming proteins (PFPs) and proteases like cathepsin-L like cysteine protease. To quantify the exoproteomic differences between the virulent and the attenuated *H*. *meleagridis* cultures, a sequential window acquisition of all theoretical spectra mass spectrometric (SWATH-MS) approach was applied. Surprisingly, results showed most of the exoproteomic differences to be of bacterial origin, especially targeting metabolism and locomotion. By deciphering such molecular signatures, novel insights into a complex *in vitro* protozoan- bacteria relationship were elucidated.

## Introduction

*Histomonas meleagridis* is a unicellular microaerophilic flagellate pathogen causing histomonosis (blackhead disease) in gallinaceous birds with a worldwide prevalence. Histomonosis in poultry is of significant importance, as it causes high mortality in turkeys and production losses in chickens [1]. The disease was well controlled in the past with the application of nitroimidazoles and nitrofurans or arsenicals for therapy or prophylaxis. However, due to new legislation in the European Union; the USA and elsewhere in the early 2000s, the use of such therapeutic or prophylactic drugs in food-producing animals became obsolete [2]. This led to the re-emergence of the disease with an increased incidence of outbreaks in poultry flocks, making histomonosis endemic again. Since its’ re-emergence, molecular data on this ‘new’ disease have been accumulating [3–9]. Molecular investigations of *H*. *meleagridis* predominantly relay on *in vitro* culture, in which the pathogen is isolated from the intestinal content of naturally infected birds and propagated in cell culture media. However, such cultures often constitute an accumulation of diverse poultry-specific caecal microbes including protozoa and bacteria, hampering histomonad specific analysis. To overcome such an impediment, a clonal culture (mono-eukaryotic) of *H*. *meleagridis* was established via micromanipulation technique, in which the parasite could be traced back to a single cell [10]. Prolonged *in vitro* sub-culturing (passaging) resulted in the attenuation of the parasite[11]. Although the establishment of the mono-eukaryotic culture facilitated protozoan specific analysis, the background of coexisting ill-defined xenic bacterial flora complicated advanced molecular studies on *H*. *meleagridis*, particularly those encompassing ‘-omics’ approaches. This necessitated the development of monoxenic culture, in which the xenic bacterial background of the mono-eukaryotic culture was exchanged with that of a single bacterial strain, the *Escherichia coli* DH5*α*, thereby generating a ‘monoxenic-mono-eukaryotic’ culture [12]. By exploiting such a defined culture, a recent study on the transcriptome of *H*. *meleagridis* disseminated a plethora of genomic information originating from 3356 unique transcripts [8]. Consequently, with the advent of a sizable volume of genomic data, further ‘-omic’ investigations on this protist were expedited. For example, comparative proteome study performed on *H*. *meleagridis* unravelled the difference between the native virulent strain and its’ mitigated attenuated strain [9].

In the current study, we focused on the role of *H*. *meleagridis* derived extracellular proteins (exoproteins/exoproteome), in particular secreted proteases. Exoproteome comprises of extracellular proteins stemming from cellular secretions (classical/non-classical mechanisms), exosome derived proteins, other export proteins and cell lysis [13]. In addition, it includes surface proteins (surfaceome), primarily membrane proteins with their domains exposed towards the extracellular space, which could have been scraped off the membrane due to experimental abrasions. Only the most stable proteins remain in exoproteome for further examination [13], hence their analysis provides invaluable information about the micro-environment of a particular cell type/biological system.

Pathogens are known to release a wide array of toxins and virulence factors including proteases into their cellular environment, which in turn can aid the development of a disease [13]. The role of exoproteins has been addressed in the context of pathological processes for a number of parasitic diseases, including trichomonosis [14,15]. Exoproteins present themselves at the interface milieu between the host and the pathogen, therefore being fundamental molecules that facilitate host-pathogen interactions [13]. It can be hypothesized that the establishment of histomonosis within the host relies on multifactorial host-parasite interactions, involving both contact-dependent and contact-independent mechanisms. Exploring virulent factors expressed in the *H*. *meleagridis* exoproteome constitutes a fundamental step towards better understanding of molecular mechanisms underlying the pathogenesis of histomonosis.

In the present study we dissected the *H*. *meleagridis* exoproteome, in order to mine for virulent factors and to obtain a comprehensive molecular outline. By applying traditional gel-based zymography and the high resolution micro-LC-ESI-MS/MS, we demonstrated the active secretion of lytic factors such as proteases by the parasite. In addition, we obtained a plethora of molecular data on extracellular environment, which at the same time, reflected the *H*. *meleagridis*-bacteria relationship.

## Materials and methods

### *Histomonas meleagridis* culture

All experiments were carried out using the monoxenic mono-eukaryotic cultures of *H*. *meleagridis* designated as *H*. *meleagridis*/Turkey/Austria/2922-C6/04. The virulent strain (*in vitro* low passage<30; LP) and the attenuated strain (*in vitro* high passage>310; HP) were utilized [12]. Parasites were passaged every 2–3 days in standard culture media, as previously described [12]. For the expansion of both virulent and attenuated *H*. *meleagridis* cultures, cells were propagated in sterile T-75 cm^2^ closed cap tissue culture flasks (Sarstedt, Wiener Neudorf, Austria) by transferring 4mL of *H*. *meleagridis* cells and 2mL of overnight grown *E*. *coli* DH5*α* culture (colony forming units (CFU): 5 x 10^8^ bacterial cells/ml) into 144mL fresh culture media and incubated at 40°C for 50-56h. Medium M199 (Gibco, Thermo Fisher Scientific, Waltham, MA, USA) supplemented with15% heat-inactivated FBS (Gibco, Thermo Fisher Scientific, Waltham, MA, USA) and 0.22% rice starch (Carl Roth GmbH + Co. KG, Karlsruhe, Germany) was used as a standard culturing medium.

### Extraction of parasite exoproteome

Both, the virulent and the attenuated *H*. *meleagridis* cultures, were maintained in sterile T-75 cm^2^ closed cap tissue culture flasks (Sarstedt, Wiener Neudorf, Austria) at 40°C for 50-56h representing the logarithmic phase of growth [16]. Following this, a protocol employing serum-free culture was executed. Briefly, the *H*. *meleagridis* cultures were transferred into sterile 50mL falcon tubes (Sarstedt, Wiener Neudorf, Austria) and centrifuged at 200x *g* for 5 min, the supernatant was discarded and the parasite pellet was gently washed using pre-warmed serum-free culture media. This step was repeated five times. Following this, the parasite cultures were pulled down to a 30mL serum-free stock aliquot, which was used to adjust the parasite concentration in the serum-free cultures. Inoculums of 30mL (1x10^6^ parasites/mL) serum—free cultures were prepared using pre-warmed serum-free culture media and 2mL overnight *E*. *coli* DH5*α* culture incubated in the same serum-free medium. The inoculums were incubated in sterile T-25 cm^2^ closed cap tissue culture flasks (Sarstedt, Wiener Neudorf, Austria) for different duration (0h, 2h, 4h, 6h, 12h, and 24h). In addition, 3 replicates of the 6h culture were prepared to be utilized in the protease inhibitor susceptibility assay and for shotgun proteomics analysis. During the incubation periods, the cell integrity was monitored under a light microscope. Cell numbers were counted using Neubauer counting chamber and cell viability was determined by trypan blue exclusion test. Throughout the experimental procedure, cells were treated gently to minimize cell lysis. Cell-free filtrates were prepared from the 30mL inoculums by differential centrifugation: 200x *g* for 5 min to remove parasites followed by 2600x *g* for 10min to remove bacteria. The supernatant was filtered through 0.22μm cellulose acetate filters (Millipore, VWR) to obtain the cell-free filtrate containing the exoproteome from the culture. For the protease inhibitor susceptibility assay and shotgun proteomics, different protease inhibitors were added at this step with concentrations mentioned below. Afterwards, the cell-free filtrate was concentrated from its’ initial 30mL volume to ~100μL. This enrichment of the sample was achieved by performing ultrafiltration, using two types of filters. Firstly, Centriprep Ultracel YM-3; 3000 NMWL (Millipore, Vienna, Austria) was used to reduce the volume from 30mL to 1mL and in the second step Amicon Ultra 0.5mL Ultracel 3K; 3000 NMWL filter (Millipore, Vienna, Austria) was implemented to obtain a final volume of ~100μL. Both types of filters were used following manufacturer’s instructions. Finally, the protein concentration of each sample was measured using Bradford Protein Assay (Fermentas, Thermo Scientific).

### 1D SDS-PAGE and 1D in gel zymography (1D-IGZ)

The electrophoretic profile of the exoproteome was analyzed by 1D SDS-PAGE (8%) and 1D substrate-copolymerized 8% PAGE (1D zymogram). For the protease *in vitro* secretion kinetic assay, 10μL of the exoproteome from each time point (0h, 2h, 4h, 6h, 12h and 24h) was loaded on the gel. For the protease inhibitor susceptibility assay, 10μg of the exoproteome from the 6h cultures (with/ without protease inhibitors) were electrophoresed. The 1D-IGZ was performed with gelatine as a substrate (0.4mg/mL). The SDS-PAGE gels were run at 100V until the bromophenol blue marker reached the anode. Following electrophoresis, the substrate gel was incubated for 1h with 2.5% Triton X-100 (Bio-Rad Laboratories GmbH, Vienna, Austria) to remove SDS and subsequently with zymogram development buffer (Bio-Rad Laboratories GmbH, Vienna, Austria) for 24 h at 37°C to activate the proteases. All gels were stained by PageBlue Protein Staining Solution (Fermentas, Fisher Scientific, Vienna, Austria). The mechanistic class of the proteases in the exoproteome was characterized by utilizing different protease inhibitors at varying concentrations. The effect of four different protease inhibitors was investigated; TLCK (Tosyl-L-lysyl-chloromethane hydrochloride) as cysteine and some serine protease inhibitor (135μM, 540μM, 1000μM), E-64 (trans-Epoxysuccinyl-L-leucylamido(4-guanidino)butane) as cysteine protease inhibitor (70μM, 140μM, 270μM), PMSF (phenylmethylsulfonyl fluoride) as serine protease inhibitor (200μM, 500μM, 1000μM) and EDTA (ethylenediaminetetraacetic acid) as metalloprotease inhibitor (200μM, 500μM, 1000μM) (SERVA Electrophoresis GmbH, Germany).

### Shotgun proteomics using micro-LC ESI-MS/MS

#### Sample preparation

The *H*. *meleagridis* exoproteomes were obtained following 6h serum-free incubation of each of three virulent and three attenuated *H*. *meleagridis* cultures. Concentrated cell-free filtrates were submitted to on-filter digestion to remove excess reagents, detergents and salts. This was followed by HPLC interfaced with micro electrospray ionization mass spectrometry (ESI-MS/MS). Briefly, the exoproteomes were treated with protease inhibitors (1000 μM TLCK, E-64, PMSF, EDTA, and cOmplete protease inhibitor cocktail tablet (Roche Applied Science, Penzberg, Germany)). Following this, 20μg protein from each samples were digested according to the standard eFASP protocol (enhanced Filter-Aided Sample Preparation) using Amicon Ultra 0.5mL Ultracel 10 K centrifugal filters (Merck Millipore, Burlington MA, USA) [17]. After washing, proteins were reduced with dithiothreitol (DTT) and alkylated with iodoacetamide. On-filter digestion was performed with trypsin/Lys-C mix (Promega, Madison, WI, USA) using 4% sodium deoxycholate for 14 hours at 37°C. Afterwards, digested peptides were recovered from the filter with three changes of 50 mM ammonium bicarbonate. Removal of sodium deoxycholate was achieved by phase transfer with ethyl acetate according to the manufacturer’s guidelines. Extracted peptides were dried down in a vacuum concentrator (Eppendorf, Hamburg, Germany). Afterwards, a C18 cleanup step using Pierce C18 Spin Columns (Thermo Fischer Scientific; Waltham, MA; USA) was performed according to the manufacturer’s manual. Dried peptides were redissolved in 5% acetonitril (ACN), 0.5% triflouroacetic acid (TFA) for sample loading. Washing was performed using 5% ACN with 0.5% TFA. Finally, peptide elution was accomplished with 70%ACN with 0.1% TFA. Eluted peptides were dried down in a vacuum concentrator (Eppendorf, Hamburg, Germany). Dried peptides were redissolved in 0.1% aqueous TFA prior to LC-MS injection (4.5 μg protein absolute in 9 μl injection volume). All samples were spiked with standardized indexed retention time reference peptides (iRT-Kit; Biognosys AG, Schlieren, Switzerland) to facilitate retention time alignment (0.8 μl iRTs added to 40 μl sample).

#### Acquisition of proteins for identification runs

Peptides were separated on an Eksigent NanoLC 425 system using a microflow pump module (Sciex, Framingham, MA, USA). Sample pre-concentration and desalting were accomplished with a 5 mm YMC-Triart C18 precolumn (500 μm inner diameter, 3 μm particle size, and 12 nm pore size) (YMC, Dinslaken, Germany). For sample loading and desalting, ultra pure LC-MS grade H2O with 0.1% formic acid (FA) was used as a mobile phase with a flow rate of 10 μl/min. Separation of peptides was performed on a 15 cm YMC-Triart C18 column (300 μm inner diameter, 3 μm particle size, and 12 nm pore size; YMC, Dinslaken, Germany) with a flow rate of 5 μl/min. The gradient started with 3% B (ACN with 0.1% FA) and increased in two steps to 25% B (68 min) and 35% (73min). It was followed by a washing step with 80% B. Mobile Phase A consisted of ultra pure H2O with 0.1% FA. For mass spectrometric analysis the LC was directly coupled to a high resolution quadrupole time of flight mass spectrometer (Triple TOF 5600+; Sciex, Framingham, MA, USA). For information dependent data acquisition (IDA runs,) MS1 spectra were collected in the range of 400–1250 m/z for 250 ms. The 40 most intense precursors with charge state 2–4, which exceeded 150 counts per second, were selected for fragmentation. MS2 spectra were collected in the range 200–1500 m/z for 50 ms. Precursor ions were dynamically excluded from reselection for 13 s. The HPLC system was operated by Eksigent Control Software version 4.2 (Sciex, Framingham, MA, USA) and the MS by Analyst Software 1.7.1 (Sciex, Framingham, MA, USA).

#### Acquisition of SWATH data

The LC parameters employed were identical to the IDA runs described above. Separation of peptides was performed on a 15 cm YMC-Triart C18 column (300 μm inner diameter, 3 μm particle size, and 12 nm pore size; YMC, Dinslaken, Germany) with a flow rate of 5 μl/min. The gradient started with 3% B (ACN with 0.1% FA) and increased in two steps to 25% B (38 min) and 35% (43min). For information independent data acquisition (SWATH runs) MS1 spectra were collected in the range of 400–1250 m/z with an accumulation time of 50 ms. Product ion spectra were collected in 70 windows in the range of 400–1250 m/z with a width of 4.8 to 323.9 Da depending on the density of precursor masses in the mass segment. For each window ions were accumulated for 50 ms.

#### Data processing

The database consisted of translated amino acid sequences originating from the 3356 non-redundant contigs of the *H*. *meleagridis* transcriptome [8] [translated: 12.10.2017], (study accession number PRJEB19109; http://www.ebi.ac.uk/ena/data/view/PRJEB19109), *E*. *coli* proteins (n = 219107) (UniProt taxonomy ID: 562, EcolX https://www.uniprot.org/taxonomy/562) [downloaded: 18.10.2017] and proteins from the common Repository of Adventitious Proteins (cRAP) (n = 117) (ftp://ftp.thegpm.org/fasta/cRAP/crap.fasta) [downloaded: 18.10.2017]. Acquired raw data were processed with ProteinPilot Software version 5.0 (Sciex, Framingham, MA, USA) for re-calibration and database searches. Mass tolerance in MS mode was set with 0.05 and 0.1 Da in MSMS mode for the rapid recalibration search, and 0.0011 Da in MS and 0.01 Da in MSMS mode for the final search. Parameters for database search were: trypsin digestion, cysteine alkylation set to iodoacetamide, search effort set to rapid ID. False discovery rate analysis (FDR) was performed using the integrated tools in ProteinPilot Software version 5.0 (Sciex, Framingham, MA, USA). Global false discovery rate was set to <1% on protein level. For quantification of proteins, IDA identification results were used to create the SWATH ion library with the MS/MS (ALL) with SWATH Acquisition MicroApp 2.0 in PeakView 2.2 (both Sciex, USA). Peptides were chosen based on a FDR rate <1%, excluding shared and modified peptides. Up to 6 peptides per protein and up to 6 transitions per peptide were used. MarkerView 1.2.1 (Sciex, USA) was used for calculation of peak areas of SWATH samples after retention time alignment and normalization using total area sums.

#### Statistical evaluation using R programming language

Statistical evaluation was performed using R programming language. cRAP proteins and proteins quantified with just one peptide were removed from Marker View raw protein list before further processing. Raw peak areas after normalization to total area sums were log2-transformed to approach a normal distribution. On a logarithmic scale, technical replicates were aggregated by arithmetic mean before application of statistical tests. Differential expression of proteins was assessed using two-tailed t-Test for independent samples for each protein. To adjust for multiple testing, the method of Benjamini and Hochberg was used to control the FDR. Protein expression was considered differential if the adjusted p-value was below α = 0.05 and the absolute fold change was at least two (fold change < −2 or > +2).

### Bioinformatics analysis

#### Gene ontology, Biological Process (BP) clustering

Using pre-existing annotations from the *H*. *meleagridis* transcriptome [8] the identified proteins were distributed into 12 categories according to their biological process.

#### Prediction of secreted proteins

In order to detect structural features indicative for protein secretion by detecting N-terminal signal peptides predicting a possible secretion, the translated amino acid sequences of the 176 *H*. *meleagridis* exoproteins were submitted to SignalP 4.1 server [18] using default parameters and organism group: eukaryotes. The SecretomeP 2.0 server [19] employing default parameters and organism group: Gram-negative bacteria, was used to predict protein secretion via non-classical pathways independent of signal peptides. Additionally, the *E*. *coli* exoproteins identified by SWATH analysis to be differentially expressed were also subjected to analysis via SignalP 4.1 and SecretomeP 2.0 server.

#### Clustering of proteins quantified by SWATH-MS analysis

Proteins quantified to be differentially expressed between the virulent and the attenuated *H*. *meleagridis* exoproteomes were clustered according to their biological process. A color coded cytoscape [20] network was generated displaying the n-fold over-expression of proteins from each *H*. *meleagridis* culture.

## Results

### Growth of *H*. *meleagridis* under serum-deprived condition

**Fig 1:**
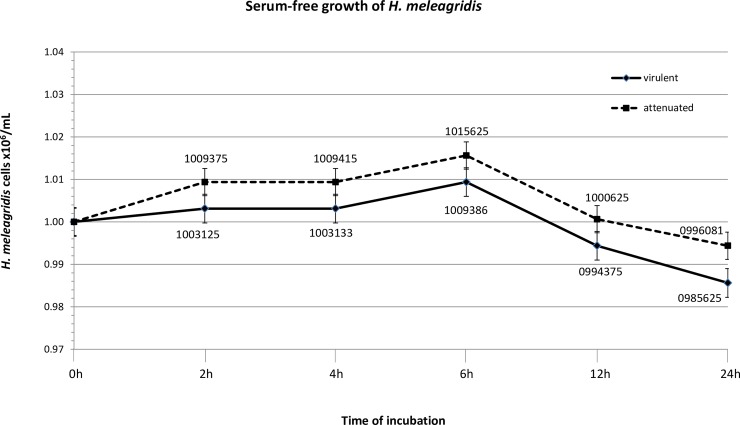
Growth curves of *H*. *meleagridis* virulent and attenuated strains under serum-free conditions. The cell viability was monitored by the trypan blue exclusion under light microscope. At any given time-point no dead cells were detected. The cell count/mL for both virulent and attenuated *H*. *meleagridis* it is shown for each time point in the growth curve and represents both total cell number and the number of viable cells.

In order to assure cell integrity, *H*. *meleagridis* cells were monitored under serum-free conditions. Under such conditions, the cell count of virulent *H*. *meleagridis* remained constant up to 6h, which was followed by a steady decline. This indicated that upon the medium exchange, cells stopped to divide but the progressive cell death started only after 6 hours of incubation in medium without serum (Fig 1). Only viable cells were observed, suggesting bursting of dead cells. The attenuated *H*. *meleagridis* displayed a slight increase in cell number within first 2 hours of incubation, which was followed by a plateau until 6 hours and steady decline in cell count after 6 hours in medium without serum (Fig 1). Since in both cultures, trophozoites started to die after 6h of incubation in serum-free medium, this time point was selected for detailed analysis of exoproteomes.

### *In vitro* secretion kinetics of proteases by *H*. *meleagridis*

**Fig 2:**
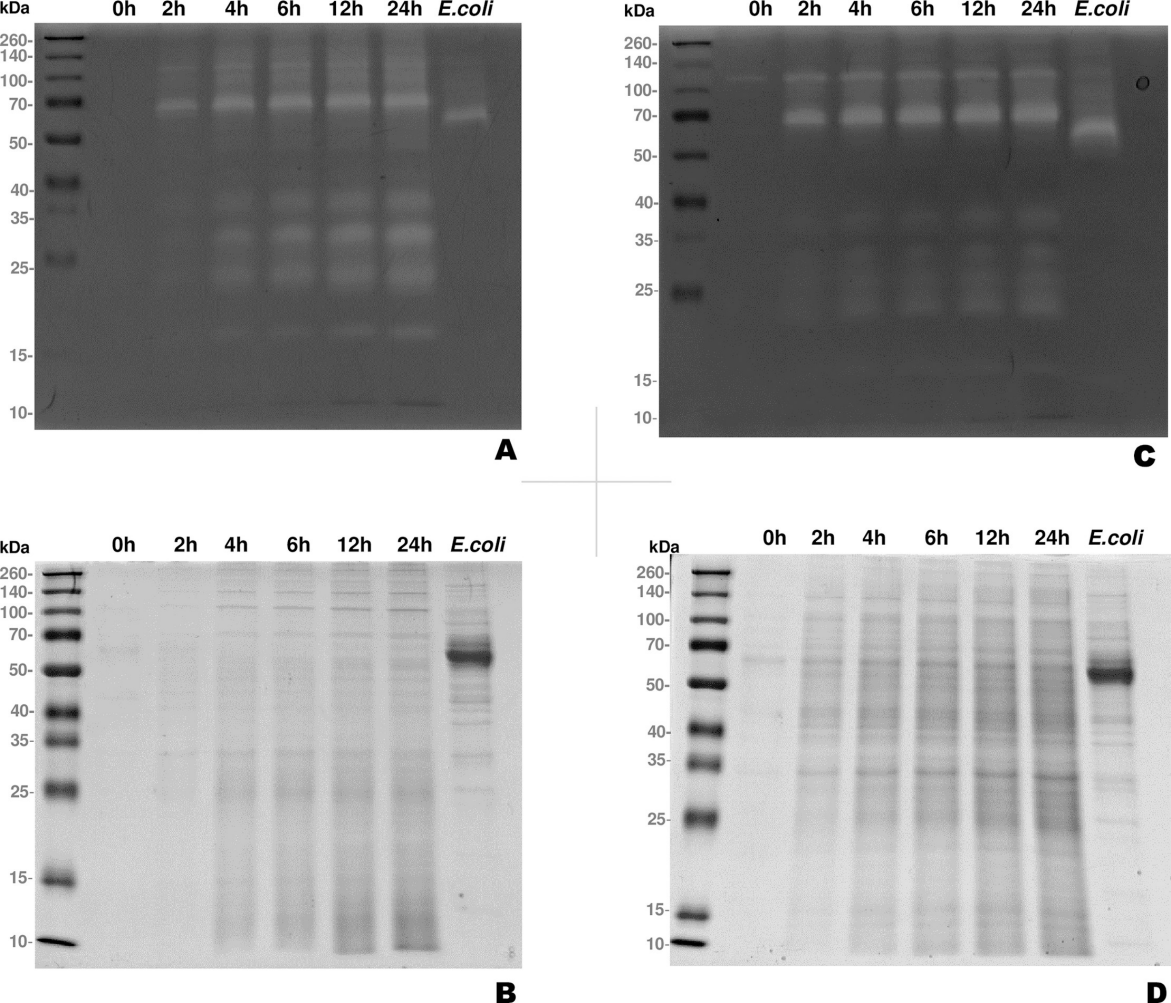
*In vitro* secretion kinetics assay demonstrating the time-dependent accumulation of proteins and proteases in the *H*. *meleagridis* exoproteome. (A) 1D-IGZ with the virulent *H*. *meleagridis* exoproteome. (B) Conventional 8% 1D SDS-PAGE gel with the virulent *H*. *meleagridis* exoproteome. (C) 1D-IGZ with the attenuated *H*. *meleagridis* exoproteome. (D) Conventional 8% 1D SDS-PAGE gel with the attenuated *H*. *meleagridis* exoproteome. *E*. *coli* 24h exoproteome serves as a control due to its’ presence in monoxenic co-cultivation.

The kinetics of proteases actively released by virulent and attenuated *H*. *meleagridis* cells into the culture media was evaluated from exoproteomes after incubating both strains in serum-free conditions for 0h, 2h, 4h, 6h, 12h, and 24h. Analysis of both exoproteomes by 1D-IGZ revealed at least six prominent protease activities corresponding to the molecular masses (MM) of approximately 17kDa, 22 kDa, 30 kDa, 36 kDa, 68 kDa and 120kDa (Fig 2). The proteases displayed a time-dependent accumulation in the exoproteomes, with their activity appearing at 2h and reaching a maximum 12h onwards (Fig 2), which indicated an active secretion. Comparison of the zymograms between the virulent and the attenuated *H*. *meleagridis* revealed a stronger protease activity of low MM proteases (< 40kDa) in the former at any time-point except 0 hours (Fig 2A and 2C). The pattern of high MM proteases did not differ between two strains. Examination of the 1D SDS-PAGE gels revealed a gradual build-up of proteins in both strains (Fig 2B and 2D).

### Protease inhibitor susceptibility assay

**Fig 3:**
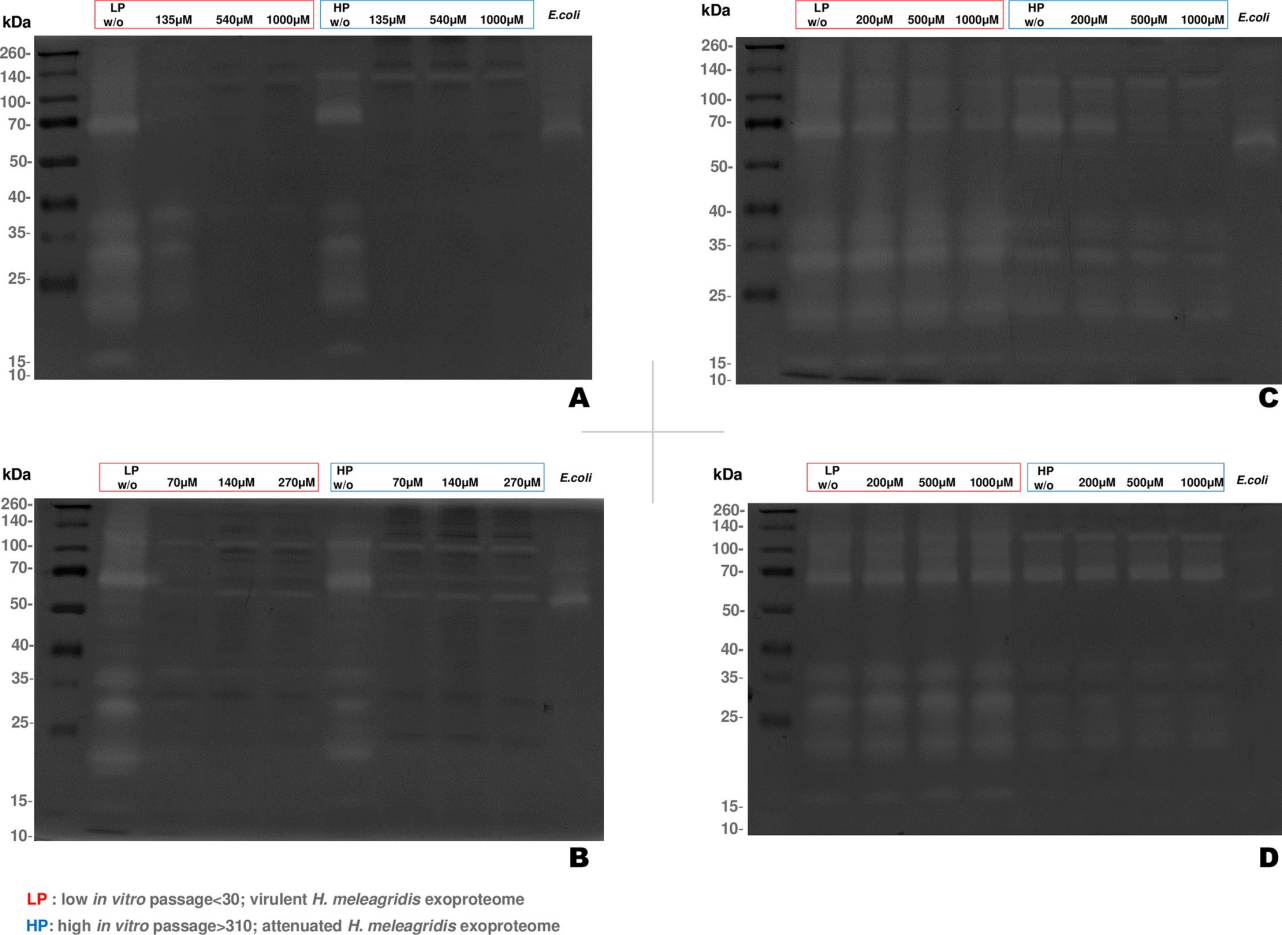
Dose dependent inhibitory effects of TLCK, E-64, PMSF and EDTA on the *H*. *meleagridis* exoproteomes. (A) 1D-IGZ demonstrating the inhibitory effect of TLCK (B), E-64 (C), PMSF and (D) EDTA. *E*. *coli* 24h exoproteome serves as a control due to its’ presence in monoxenic co-cultivation.

The mechanistic class of proteases in the *H*. *meleagridis* exoproteome was characterized based on their susceptibility to different protease inhibitors. The exoproteomes from both, virulent and attenuated *H*. *meleagridis* cells, were treated with the protease inhibitors: TLCK, E-64, PMSF and EDTA and subjected to 1D-IGZ analysis. The susceptibility of six protease bands at MM of ~ 17kDa, 22 kDa, 30 kDa, 36 kDa, 68 kDa and 120kDa was most evident (Fig 3). A dose-dependent manner of protease inhibition was observed, with the effect of TLCK being most profound, followed by E-64, PMSF and EDTA. In addition, strain dependant variations in the minimal concentration of inhibitor needed to suppress the protease activity were noticed. For TLCK, a potent dual inhibitor of cysteine and serine proteases, a concentration of 135μM was sufficient to subdue the majority of the protease activity in the attenuated *H meleagridis* exoproteome (Fig 3A). In contrast, a higher concentration of 540μM was required to achieve similar effect in the virulent exoproteome (Fig 3A). The inhibitor E-64, which selectively inhibits cysteine proteases, showed a similar inhibitory effect than TLCK. Again, much lower concentration (70μM) was sufficient to subdue the majority of the protease activity in the attenuated exoproteome, in contrast to its’ virulent counterpart that needed 270 μM E-64 for suppression of protease activity (Fig 3B). To determine the distribution of serine proteases, inhibitor PMSF was applied. In general, the effect of PMSF on the exoproteome of either *H*. *meleagridis* strain was almost non-existent (Fig 3C). Exceptional to this is the proteolytic activity at ~ 68kDa, which exhibited susceptibility to the PMSF in the attenuated exoproteome at 500 and 1000μM. In contrary to this, even with the highest concentration applied (1000μM) the virulent exoproteome was not influenced by PMSF. The distribution of metalloproteases was assessed by addition of EDTA, however its’ inhibitory effect remained largely inert to exoproteomes of both strains (Fig 3D).

### Shotgun proteomics to identify proteins in the *H*. *meleagridis* exoproteome

**Fig 4:**
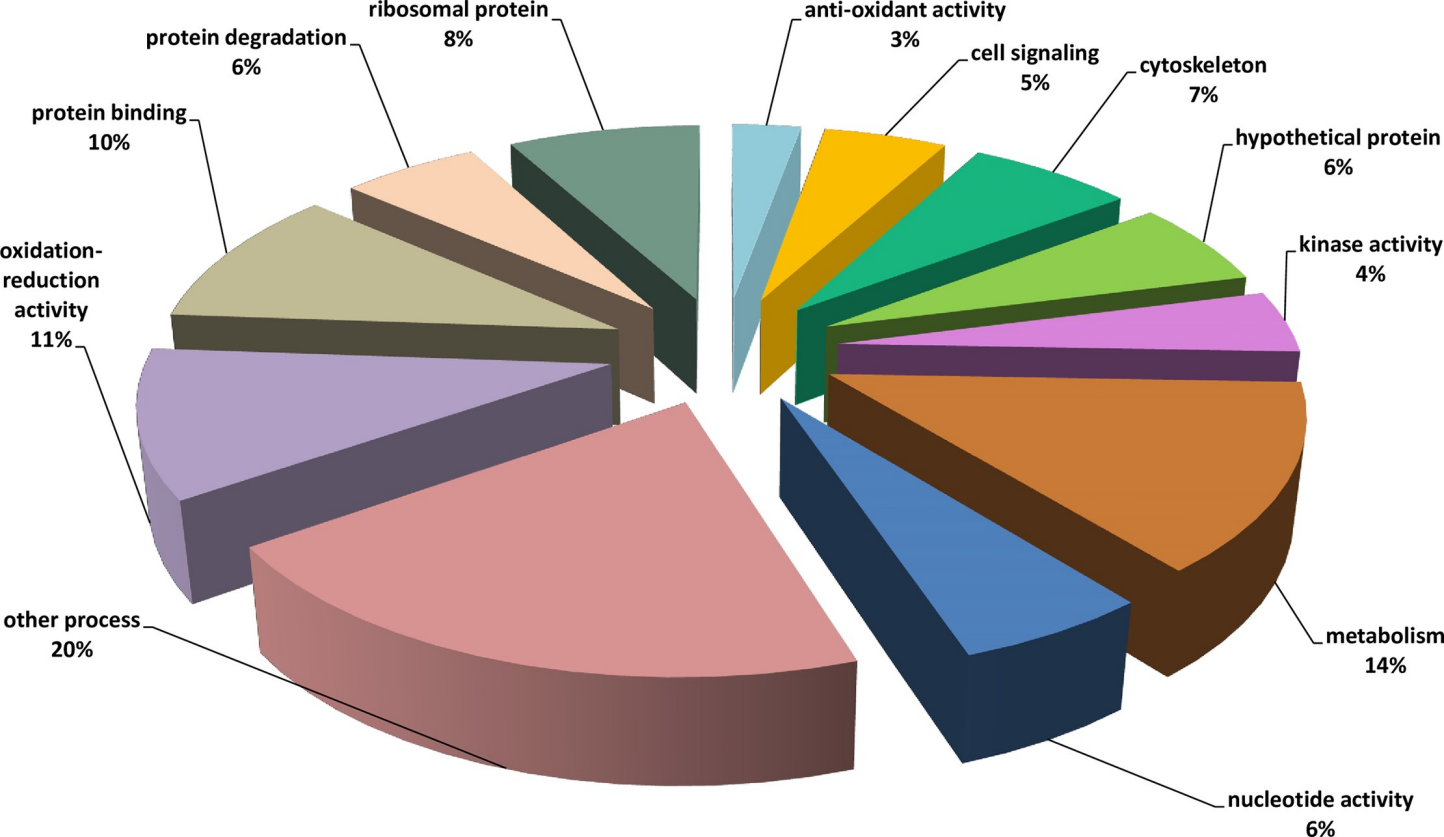
Pie-chart displaying the proteins identified in the *H*. *meleagridis* exoproteome sorted according to their biological process. The proteins were categorized into 12 groups termed as: anti-oxidative stress, cell signalling, cytoskeletal, hypothetical protein, kinase activity, metabolism, nucleotide activity, other processes, oxidation-reduction activity, protein binding, protein degradation and ribosomal protein.

Following ESI-MS/MS, the resulting peptides were analysed and mapped to the *H*. *meleagridis* reference transcriptome (3356 non-redundant transcripts) [8] identifying a total of 176 proteins with at least two peptides displaying confident ion scores, at false discovery rate (FDR) of <1% (S1, S2 and S3 Tables). The 176 proteins were grouped into 12 categories based on the gene ontology, biological process (BP) (Fig 4). The 176 proteins identified in the exoproteome represented 5.2% of the *H*. *meleagridis* transcriptome. The SignalP algorithm detected 17 proteins possessing the N-terminus signal peptide, whereas SecretomeP 2.0 algorithm predicted 37 proteins to be secreted independently of signal peptides via non-classical pathways (S1 Table). In addition to *H*. *meleagridis* proteins, the analysis identified 647 *E*. *coli* proteins (S2 and S3 Tables).

### Differentially expressed proteins quantified by SWATH-MS analysis

**Fig 5:**
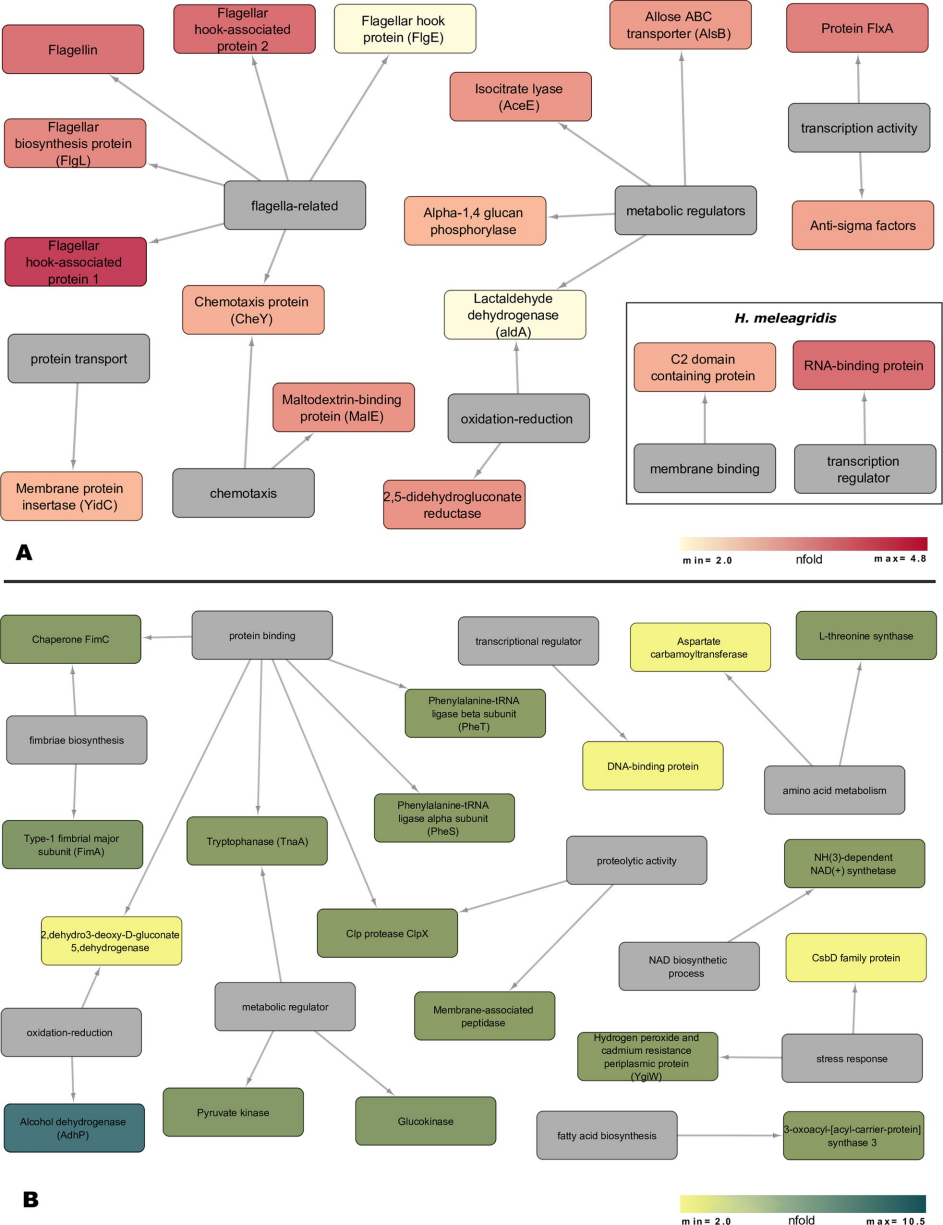
Protein function network of up-regulated proteins detected by the SWATH-MS approach (≥ 2-fold and p-value < 0.05). (A) *H*. *meleagridis* virulent exoproteome, (B) *H*. *meleagridis* attenuated exoproteome. The source nodes are categorized according to biological process of the proteins. The colour-coding is based on the n-fold over-expression data associated with each significantly differentially expressed protein (Tables 1 and 2). The network layout was manually determined. Over expressed proteins in virulent histomonads (A) are displayed in small inserted figure.

Exoproteomes from three biological replicates of virulent and attenuated *H*. *meleagridis* cultures were compared by SWATH-MS to determine the difference in protein expression. The SWATH data from two injections for each replicate were analysed by ProteinPilot Software version 5.0 (Sciex, USA). Data analysis (p-value < 0.05; fold change < −2 or > +2) resulted in the identification of 35 differentially expressed proteins (Fig 5). Out of the 35 proteins, two proteins, a C2 domain containing protein and a RNA binding protein, were mapped exclusively to the virulent *H*. *meleagridis* (Table 1). The remaining 33 proteins were mapped to *E*. *coli*, with 17 proteins over-expressed in the exoproteome of the virulent (Table 1) and 18 in the attenuated culture (Table 2). The 17 *E*. *coli* proteins identified as over-expressed in the virulent *H*. *meleagridis* background included proteins involved in bacterial motility, carbohydrate membrane transport and carbohydrate metabolism (Table 1), whereas the 18 *E*. *coli* proteins over-expressed in the attenuated exoproteome can be related to the stress response toward the parasite (Table 2).

**Table 1:** Summary of up-regulated proteins detected by the SWATH-MS method in the virulent *H*. *meleagridis* exoproteome (≥ 2-fold and p-value < 0.05).

Species-Contig ID/Accession Number^a^	Protein identity^b^	n-fold upregulation^c^	FDR-adjusted P value^d^	No. of confident peptides^e^	SignalP^f^	Secre-tomeP^g^
***E*. *coli***						
J7R3D6_ECOLX	Flagellar hook-associated protein 1	4.8	0.005	6		X
A0A1Y2XUE9_ECOLX	Flagellar hook-associated protein 2	3.8	0.001	6		X
Q53ZW4_ECOLX	Flagellin	3.7	0.002	6		X
A0A0J2A7V6_ECOLX	Protein FlxA	3.4	0.003	5		X
W8SPA8_ECOLX	Flagellar biosynthesis protein (FlgL)	3.2	0.002	6		X
E2D2R3_ECOLX	2,5-didehydrogluconate reductase	3.0	0.001	6		
C3SHQ8_ECOLX	Maltodextrin-binding protein (MalE)	3.0	0.001	6	X	X
E2QJ37_ECOLX	Isocitrate lyase (AceE)	2.9	0.008	5		
W8SXT6_ECOLX	Allose ABC transporter (AlsB)	2.8	0.003	6	X	X
J7Q703_ECOLX	Anti-FliA (Anti-sigma) factor also known as RflB protein	2.6	0.002	5		X
C3T5G7_ECOLX	Chemotaxis protein (CheY)	2.4	0.014	2		
W8T8E9_ECOLX	Alpha-1,4 glucan phosphorylase	2.2	0.006	6		
W8U2R6_ECOLX	Membrane protein insertase (YidC)	2.2	0.007	3		X
W8U164_ECOLX	Aldehyde dehydrogenase	2.0	0.003	6		
J7Q705_ECOLX	Flagellar hook protein (FlgE)	2.0	0.049	2		X
***H*. *meleagridis***						
HAGI01001912	RNA-binding protein	3.8	0.002	2		
HAGI01001509	C2 domain containing protein	2.4	0.003	4		

^a^Contig identification number (Contig ID) corresponds to the respective transcript in the *H*. *meleagridis* reference transcriptome ^7^. Accession number corresponds to the respective ID in the *E*. *coli* database (UniPort taxonomy ID: 562, EcolX https://www.uniprot.org/taxonomy/562).

^b^Protein identity refers to the annotation of respective Contig ID and Accession number.

^c^The n-fold over-expression of each protein data as retrieved from the SWATH-MS analysis.

^d^Cut-off p-value by which protein expression was considered differential.

^e^The number of confident peptides by with each protein was detected.

^f^Prediction of N-terminal signal peptides by SignalIP 4.1.

^g^Prediction of protein secretion via non-classical by Secretome P 2.0 server.

**Table 2:** Summary of up-regulated proteins detected by the SWATH-MS method in the attenuated *H*. *meleagridis* exoproteome (≥ 2-fold and p-value < 0.05).

Species-Contig ID/Accession number^a^	Protein identity^b^	n-fold upregulation^c^	FDR-adjusted P value^d^	No. of confident peptides^e^	SignalP^f^	Secre-tomeP^g^
***E*. *coli***						
W8SZ36_ECOLX	Alcohol dehydrogenase (AdhP)	10.5	0.003	6		
Q9F0R7_ECOLX	Type-1 fimbrial major subunit (FimA)	3.3	0.002	2	X	X
J7R6X0_ECOLX	Glucokinase	3.1	0.014	5		
Q2TL68_ECOLX	Pyruvate kinase	3.0	0.003	6		
Q643Q7_ECOLX	Chaperone FimC	2.5	0.001	6		X
C3TRQ2_ECOLX	L-threonine synthase	2.4	0.025	4		
A0A136XMV7_ECOLX	Phenylalanine-tRNA ligase alpha subunit (PheS)	2.3	0.004	6		
W8T0N9_ECOLX	Tryptophanase (TnaA)	2.3	0.003	6		
A0A0M1UG24_ECOLX	Clp protease ClpX	2.3	0.003	3		
W8SZ23_ECOLX	Membrane-associated peptidase	2.2	0.003	6	X	X
W8TE29_ECOLX	NH(3)-dependent NAD(+) synthetase	2.2	0.021	6		
J7R1N6_ECOLX	Phenylalanine-tRNA ligase beta subunit (PheT)	2.2	0.003	5		
C3SUG2_ECOLX	Hydrogen peroxide and cadmium resistance periplasmic protein (YgiW)	2.2	0.003	3	X	X
C3TDZ2_ECOLX	3-oxoacyl-[acyl-carrier-protein] synthase 3	2.1	0.017	5		
W8TW10_ECOLX	2-dehydro-3-deoxy-D-gluconate 5-dehydrogenase	2.0	0.007	6		
C3SI42_ECOLX	DNA-binding protein	2.0	0.012	6		X
C3SF53_ECOLX	Aspartate carbamoyltransferase	2.0	0.021	4		
D3Y3A6_ECOLX	CsbD family protein	2.0	0.024	2		X

^a^Contig identification number (Contig ID) corresponds to the respective transcript in the *H*. *meleagridis* reference transcriptome ^7^. Accession number corresponds to the respective ID in the *E*. *coli* database (UniPort taxonomy ID: 562, EcolX https://www.uniprot.org/taxonomy/562).

^b^Protein identity refers to the annotation of respective Contig ID and Accession number.

^c^The n-fold over-expression of each protein data as retrieved from the SWATH-MS analysis.

^d^Cut-off p-value by which protein expression was considered differential.

^e^The number of confident peptides by with each protein was detected.

^f^Prediction of N-terminal signal peptides by SignalIP 4.1.

^g^Prediction of protein secretion via non-classical by Secretome P 2.0 server.

## Discussion

The present study focused on investigating the exoproteomes of *H*. *meleagridis*, derived from virulent and attenuated cells cultivated in serum-free conditions. Since serum-free conditions were a necessary step to prevent the unspecific results from serum proteins [21], the cellular integrity of *H*. *meleagridis* was monitored for any adverse effects arising from such conditions[22]. Despite showing no active growth under serum-free conditions [23], slight differences between the growth of two *H*. *meleagridis* cultures were noticed. In contrast to virulent histomonads, the cell count of attenuated parasites slightly increased within first two hours of incubation. This could be due to the fact that at the start of the serum-free experiment, cells were taken from the mid logarithmic phase and just continued their programmed cell division. Attenuated *H*. *meleagridis* cells are well adapted to *in vitro* growth conditions in complete medium, showing higher cell numbers as compared to the virulent parasites [16]. It is therefore plausible that attenuated histomonads needed slightly longer to completely cease their growth. This observation is coherent with the recent study reporting *in vitro* phenotypic differences between the two strains, where drastic changes in temperature and pH levels that can account as ‘stress factors’, resulted in pronounced effects in the virulent *H*. *meleagridis* culture [16].

The exoproteomes, used in the experiments with protease inhibitors and for proteomic analysis, were extracted from cultures incubated for 6h. At this time point, drastic cell death could not be detected for both strains and sufficient protein for analysis could be harvested. Therefore, it can be anticipated that the majority of proteins originated from an active secretion, as demonstrated by the *in vitro* secretion kinetics of proteases by *H*. *meleagridis*. Furthermore, since discrimination between proteins sequestered inside extracellular vesicles from their soluble counterparts was not performed, a sizable proportion of the proteins derived from exosomes should be expected.

The biological role of proteases has been extensively discussed in the context of parasitic protozoa, including their role in pathogenesis [24–26]. Applying 1D-IGZ investigations, a wide spread distribution of proteases was detected ranging from low to high MM, similar to results obtained for *Trichomonas gallinae* and *Trichomonas vaginalis* [27,28]. It can be predicted that these molecules play an important role in the *H*. *meleagridis* micro-environment, since their secretion was noticed already after 2h of incubation. The existence of these lytic factors in the exoproteome of *H*. *meleagridis* supports the notion of nutrient acquisition by attacking the co-cultivating *E*. *coli*.

Inhibitory effects of TLCK and E-64 were the most profound, suggesting that the majority of *H*. *meleagridis* extracellular proteolysis is carried out by cysteine proteases. This is not surprising given the fact that transcripts encoding for cysteine proteases were the most over-represented protease family in the *H*. *meleagridis* transcriptome [8]. The results of the TLCK and E-64 inhibition tests demonstrate that the activity of cysteine proteases within the virulent *H meleagridis* exoproteome displays higher robustness than the one within the attenuated exoproteome. Higher concentrations of TLCK and E-64 were required to subdue the protease activity of virulent exoproteome, showing reduced sensitivity to the inhibitors in contrast to its’ attenuated counterpart. Cysteine proteases have been implicated in virulence of a number of pathogens, including parasitic protozoa such as trichomonads [29–31]. For instance, from the study on *T*. *gallinae* [27] one could easily emphasize the association of cysteine proteases with the pathogenicty, as the addition of TLCK and E-64 averted the cytotoxic effect of the parasites’ exoproteome on LMH cells. Mass spectrometric investigations of the *T*. *gallinae* proteases revealed the action of a particular clan of cysteine protease to be the major cytotoxic molecule; the C1 family cathepsin L-like cysteine protease [27]. Likewise, research on the human pathogen *T*. *vaginalis* highlights the role of its’ cysteine proteases in the context of pathogenesis [28,32]. Particularly cathepsins have been demonstrated to facilitate the parasites’ adherence to host cells and induce cellular damage [33–35].

The shotgun proteomics analysis of the *H*. *meleagridis* exoproteome identified three cysteine proteases represented by C1 family cathepsins and C13 family legumains. The majority of the cysteine proteases identified here had a low MM range (30–40 kDa region), with two cathepsins and one legumain protease displaying a theoretical MM of 34.88 kDa, 35.65 kDa and 47.25 kDa, respectively. Correlating this data to the 1D-IGZ results, it seems that the majority if not all of the proteolytic activities up to ~ 40 kDa in the *H*. *meleagridis* exoproteome, stem from the cysteine proteases. Discrepancy of few kDa between the theoretical and actual MM as observed in here is not unusual and discrepancies of up to 13 kDa have been reported [36]. Proteins often undergo chemical changes due to posttranslational modifications such as glycosylation and ubiquitination/sumoylation [37], which can shift their mobility pattern during electrophoresis. Alternatively, the reported *H*. *meleagridis* transcripts may not be of full length.

The *H*. *meleagridis* exoproteome remained predominantly inert to PMSF, suggesting a sparse distribution of serine proteases. In agreement with this, the shotgun proteomic approach identified only a single serine protease in the exoproteome with one confident peptide (theoretical MM:18.68kDa, S2 Table). Interestingly, the only protease activity which displayed some susceptibility to PMSF during 1D-IGZ analysis was the protease at ~68kDa in the exoproteome of attenuated *H*. *meleagridis*. The activity of this protease was more robust in the exoproteome of virulent *H*. *meleagridis* in which it persisted even with the addition of 1000μM PMSF. As shotgun proteomics analysis was negative for higher MM serine proteases, the effects of PMSF might be due to the presence of other class of proteases. In addition to serine proteases, PMSF also inhibits clan C1 family peptidases to certain extent, the only class of cysteine protease susceptible to this inhibitor [38]. Based on the sensitivity of the ~68kDa protease band to TLCK and E-64, it seems very likely that this is a cathepsin, which is also susceptible to higher concentrations of PMSF.

The shotgun proteomic approach identified metalloproteases belonging to the M20 family T- like metallopeptidase, M24 family aminopeptidase and M49 family metalloproteases. However, during the 1D-IGZ analysis, the *H*. *meleagridis* exoproteome remained overall inert to EDTA, suggesting negligible presence of metalloproteases. Such a discrepancy may arise from amino acid sequence homology analysis, which identified several of these proteins as metalloproteases. However, they might also represent non-peptidase homologues, which carry out metal binding activity instead of actual proteolysis[39]. An alternative explanation might be that the metalloproteases identified here are inactive zymogens, which require specific modifications under appropriate conditions to become active.

Unlike the differences in proteolytic intensities between the virulent and the attenuated *H*. *meleagridis* exoproteome noticed by 1D-IGZ analysis, the applied SWATH-MS approach could not quantify any differences in protease expressions between the two strains. It appears that the lack of protease inhibitors in protease secretion kinetics assays, as opposed to the 1D-IGZ inhibitor assays and the mass spectrometric analysis, actually demonstrated the potency of proteases in the virulent strain rather than its’ expression levels. This is corroborated by the results of protease inhibitor susceptibility assay, in which the lowest effective concentration of all tested inhibitors was much higher for the virulent as opposed to the attenuated exoproteome.

The shotgun proteomic approach identified 176 proteins with at least two confident peptides, which accounts for 5.2% of the *H*. *meleagridis* transcriptome. By utilizing the pre-existing annotations from the *H*. *meleagridis* transcriptome database [8], the proteins were classified into twelve categories based on their biological process. *In silico* analysis by SignalP and SecretomeP algorithms, showed that proteins secreted by non-classical pathways are dominant in the exoproteome, although the very small proportion of proteins from a sporadic cell lysis cannot be excluded. The actual results suggest that *H*. *meleagridis* predominantly exports proteins by means of non-classical pathways in contrast to traditional signal peptide based motifs, which might prove to be advantageous to the parasite. Proteins not dictated by premeditated export signal peptide motifs might actually carry out two unrelated functions, within the cytoplasm and in the extracellular milieu, demonstrating a moonlighting property [19]. This could facilitate *H*. *meleagridis* survival and/or virulence by maximizing its’ proteomic functions.

The identification of actin and several gylcolytic enzymes, such as enolase, fructose bis-phosphate aldolase (FBA), glyceraldehyde-3-phosphate dehydrogenase (GAPDH), and triose-phosphate isomerase (TPI), in the *H*. *meleagridis* exoproteome is particularly intriguing. Common traits of all these proteins are their alternative localization on the parasite’s surface and the capacity to bind plasminogen [40]. The recent comparative proteome studies of *H*. *meleagridis*, identified actin, enolase, FBA and GAPDH as up regulated by virulent histomonads [9, 41]. However, this only reflected spots localized on positions that differed to the bulk of both proteins, indicating possible post-translational modification(s) and/or truncation(s). Both studies suggested that such modified actin, enolase, GAPDH and FBA molecules might be components of alternative mechanism (moonlighting) unrelated to cytoskeleton and carbohydrate metabolism. Similar observations, involving the up regulation in the virulent strain and alternative positions on 2D-proteome map, were also reported for *T*. *vaginalis* [42,43] Binding of plasminogen to these parasites’ surface exposed molecules, is a crucial step in the activation of plasminogen to plasmin, a serine protease [44]. Using this mechanism, the parasite interacts with the fibrinolytic system of the host, which is involved in degradation of fibrin and extracellular matrices [44]. The presence of actin, enolase, GAPDH, FBA and TPI molecules in the exoproteome of *H*. *meleagridis* could therefore be related to their tentative plasminogen-binding role. Considering that the dissemination of *H*. *meleagridis* from caecum to liver and other organs occurs via blood, a mechanism involving the interaction with host’s fibrinolytic system might be of great importance. Beside potential plasminogen binding proteins, another group of moonlighting proteins, such as adhesins AP-33, AP-51, and AP-65, was identified in the *H*. *meleagridis* exoproteome. Classically localized in hydrogenosomes as metabolic enzymes, these proteins have been reported to be surface attached, mediating cytoadherence to host cells [45]. An earlier study, reported only the *H*. *meleagridis* AP-33 and its’ dual localization, both in the hydrogenosomes and on the cell surface, whereas AP-65 could only be detected within the hydrogenosome [7]. The results presented here, suggest that besides of being intracellular, AP-33, AP-51 and AP-65 are also localized on the cell surface or secreted by *H*. *meleagridis*, which could reinforce their moonlighting property.

Data mining also revealed the presence of several lytic molecules in the *H*. *meleagridis* exoproteome particularly surfactant B/saposin-like proteins, zinc dependent phospholipase C and N-acetylmuramoyl-L-alanine amidase. Most of these lytic molecules operate by attacking target cell membrane and compromising cellular integrity [46–49]. In the present context, these molecules are most likely exploited by *H*. *meleagridis* to launch assaults against co-incubating bacteria, a function they might display *in vivo* against host cells.

The SWATH-MS approach was applied to obtain a quantitative proteome expression between the virulent and the attenuated *H*. *meleagridis* cultures. The majority of the differentially expressed proteins were of *E*. *coli* origin whose detection was not surprising due to the monoxenic co-cultivation with *E*. *coli* DH5*α*. The applied statistical parameters filtered only two *H*. *meleagridis* proteins as differentially expressed, a C2 domain containing protein and a RNA-binding protein, both over-expressed in the virulent exoproteome. Both proteins have a wide spectrum of functions [50,51]. The C2 domain is a membrane binding motif found in a broad and diverse set of eukaryotic proteins [50]. The majority of the C2 domains function as phospholipid binding moieties relying on calcium (Ca^2+^), hence sometimes referred to as a Ca^2+^ dependent lipid binding domains [52]. Lipid-binding domains (LBDs) can engage in a wide set of molecular functions, such as membrane anchoring of the cytoskeleton [53,54]. The identification of such a protein in the exoproteome stems most likely from experimental conditions, which introduced a removal of an extracellular domain of such membrane bound protein. Its’ up-regulation in the virulent *H*. *meleagridis*, might indicate a possible cytoadherence mechanism that involves the interaction of the parasite’s C2 motif protein and host cells.

The RNA-binding proteins engage in the formation of ribonucleoprotein complexes by binding to sequence/structural motifs in the RNA, which can alter the fate of that RNA molecule[51]. The presence of parasite derived extracellular RNA (exRNA) recognition motif can thus have far-reaching implications. Such proteins can serve as ‘traps’ targeting host cell exRNA, which upon binding can aggravate host susceptibility. It is noteworthy to point that, exRNAs particularly in extracellular vesicles are an important source of cellular communication both in protists and multi-cellular organisms [55,56].Therefore one can hypothesize on the implication of *H*. *meleagridis* derived exRNA-binding motif in the disruption of the host cell signalling.

It is intriguing to consider why only two *H*. *meleagridis* proteins were detected as significantly over-expressed. One possible explanation would be that the serum-free conditions provoked a stress effect and by that possibly reduced the overall metabolism of both *H*. *meleagridis* strains which lead to a minimization of excreted proteins. Additionally, since the attenuated *H*. *meleagridis* strain originated from the virulent by prolonged *in vitro* cultivation, both strains are genetically very similar [11]. The recent *H*. *meleagridis* proteome analysis study somewhat argues for the first hypothesis, since the use of identical *H*. *meleagridis* strains and the SWATH MS approach identified 42 differentially expressed *H*. *meleagridis* proteins [41]. However, beside the fact that in this study the medium for cultivation of parasites contained serum, the analysed protein fraction was different and contained endogenous proteins. Taken together, it seems that even though the serum-free stress conditions have to be considered, the bulk of variations between the two *H*. *meleagridis* strains in *in vitro* conditions is endogenous

The majority of differentially expressed proteins identified in the present study were of *E*. *coli* origin. This demonstrates the rapid change in the prokaryote’s gene expression which is dependent on the parasite’s phenotype. At the same time, it indicates a strong interaction between *H*. *meleagridis* and bacteria. This relationship is particularly thought-provoking, as the bacteria are not only required for proliferation of *H*. *meleagridis in vitro*; but their presence in the intestines of host birds is essential for successful infection [57]. Furthermore, different bacterial strains mediate diverse stimulatory effects on *H*. *meleagridis* growth *in vitro* [12]. A recent comparative proteomics study supports the idea of interdependence and complex interactions between *H*. *meleagridis* and bacteria [9]. However, the nature of the *H*. *meleagridis* -prokaryote relationship is still unresolved.

The *E*. *coli* proteins over-expressed in the *H*. *meleagridis* virulent background were involved in the flagellar motility system or carbohydrate uptake. It seems that the low glucose environment of Medium 199 induced the uptake mechanisms of carbohydrate sources, such as maltodextrin or D-allose. The MalE protein was also shown to be important in glucose transport under glucose-limited conditions [58]. Aside from this, a considerable number of proteins involved in flagellar motility were up-regulated, which presumably facilitated bacteria in accessing a more favourable environment [59]. The medium that was used in the present study contains low glucose and is supplemented with rice starch as an additional carbohydrate source. Since *E*. *coli* is unable to use rice starch due to the absence of endogenous amylolytic enzymes [60], glucose remains the only possible carbohydrate source. In contrast, *H*. *meleagridis* is able to hydrolyse starch molecules [8], and could potentially contribute to the increase of extracellular glucose. However, it is likely that the serum-free medium caused the virulent *H*. *meleagridis* to severely minimize/or stop metabolizing, since even in optimal conditions, this strain of the parasite is not well adapted to *in vitro* growth and shows much lower metabolic rates as compared to the attenuated parasite [9,16].

In contrast to the virulent culture, *E*. *coli* proteins up regulated during co-cultivation with attenuated *H*. *meleagridis* indicate a stress response toward the parasite. This particularly concerns ATP-dependent Clp protease (ClpX), hydrogen peroxide and cadmium resistance periplasmic protein (YgiW) and CsbD family protein, which were all described to be associated with stress response [61–63]. As adapted to the *in vitro* growth, the attenuated *H*. *meleagridis* seems to cope more successfully with the serum-free conditions than its’ virulent counterpart [9,16]. It is possible that attenuated *H*. *meleagridis* phagocytised co-cultivating *E*. *coli* more effectively than the virulent parasite, which in turn resulted in the up-regulation of *E*. *coli* stress proteins. In addition, the up-regulation of the tryptophanase (TnaA) together with proteins involved in the formation of type -1 fimbriae (FimA and FimC), detected in the attenuated *H*. *meleagridis* exoproteome, can be related to the interaction with the parasite. Type-1 fimbriae are most likely involved in the direct contact with the parasite, since they enable the bacteria to bind specific receptor structures [64]. However, the up-regulation of the TnaA protein might indicate a potential defence mechanism of *E*. *coli* towards phagocytosis by the parasite. The tryptophanase is an enzyme responsible for the inter-conversion between tryptophan and indole [65]. The more favoured product of the reaction, the indole, has been proposed to act as an extracellular signalling molecule influencing a variety of biological processes, even playing role in inter-species communication signals [66]. In *Vibrio cholerae*, active tryptophanase and the presence of indole are related to grazing resistance against the phagocytic eukaryote *Dictyostelium discoideum* [67]. Whether a similar mechanism is employed by *E*. *coli*–histomonad interaction has to be investigated in more detail. However, considering that a reduction of *E*. *coli* during co-cultivation with *H*. *meleagridis* was not observed [12], it is possible that the prokaryote employs some protective mechanism to protect from phagocytosis by the parasite. Finally, the strikingly high rate of overexpression of *E*. *coli* AdhP alcohol dehydrogenase in the exoproteome of attenuated *H*. *meleagridis* is especially intriguing. Unlike the more common alcohol dehydrogenase AdhE, the AdhP is an ethanol-inducible dehydrogenase whose expression is not linked to anaerobic conditions [68]. Its’ up-regulation in the attenuated *H*. *meleagridis* culture indicates an increased concentration of ethanol in the media, whose likely contributor is the parasite itself. This situation resembles low-iron-dependent changes described for *Tritrichomonas foetus*, in which the pyruvate metabolism is shifted to the cytoplasm resulting in the production of ethanol and some glycerol [69]. The existence hydrogenosomal iron-sulphur proteins, such as PFOR, ferredoxin and iron-hydrogease in *H*. *meleagridis*, indicates the requirement for iron supplementation in order to maintain normal hydrogenosomal metabolism and energy production [8]. In *in vitro* conditions the serum represents an important iron source that is absent in the current experiment. Therefore, it is likely that due to the lack of iron, the majority of hydrogenosomal metabolism is inhibited and pyruvate is utilized in the cytoplasm, resulting in the production and subsequent secretion of the ethanol [69]. The *E*. *coli* AdhP enzyme, in turn, converts ethanol to acetaldehyde which can again be used by the protozoan cytoplasmic fermentive metabolism, releasing the reduced NADP+ molecules and maintaining redox balance. When compared to the virulent *H*. *meleagridis* culture, there seems to be no elevation in ethanol concentration in the media. Based on the serum-free growth and previous reports [9,16], it is likely that the virulent parasite, due its’ overall *in vitro* maladaptive state, dramatically slowed down its’ metabolism just for mere survival purpose. Overall, this underlines the complexity of the bacteria-parasite interaction.

## Conclusions

The *in vitro* secretion kinetics assay of proteases demonstrated the significance of these lytic molecules in *H*. *meleagridis* extracellular milieu, showing a time-dependent secretion from as early as 2 hours post-incubation. Comparative 1D-IGZ analysis between the virulent and the attenuated *H*. *meleagridis*, displayed the robustness of proteases secreted by the virulent strain. The protease inhibitor susceptibility assay revealed cysteine proteases to be the predominant lytic molecules in the *H*. *meleagridis* exoproteome, with the higher potency in cultures of virulent parasites. The comprehensive analysis of the *H*. *meleagridis* exoproteome using high-resolution proteomics identified a pool of 176 proteins, providing an extensive molecular overview of the *H*. *meleagridis* extracellular milieu and indicating its’ dynamic micro-environment. The quantitative exoproteome analysis of the virulent and the attenuated *H*. *meleagridis* cultures displayed differences in proteins of co-cultivating *E*. *coli*, which in turn reflected the protozoa-bacteria interaction for the first time at the molecular level. Differences between *H*. *meleagridis*-derived exoproteins were almost non-existent, suggesting that in *in vitro* conditions the majority of proteomic alterations are endogenous, as previously observed in our comparative proteome studies. Overall, the present study investigating the exoproteome of *H*. *meleagridis* provides an attractive avenue for further research, hypothesis testing and comparative analysis of strains with different biological properties.

## Supporting information

S1 Table List of proteins identified in the *H. meleagridis* exoproteome. The list contains *H*. *meleagridis* specific proteins identified in the exoproteome with ≥2 confident peptides in at least 3 replicates. Information on SignalP and SecretomeP data, number of confident peptides, the % of protein sequence coverage (>95% confidence) and the number of ambiguous accessions is given. The total ProtScore means the score with all peptides (unique and shared). The Unused ProtScore is the score using only the unique peptides. Term “ALL” represents combined data for all 6 replicates. The LP1, LP2, and LP3 are 3 replicates of the *H*. *meleagridis* virulent strain (low passage, 28x). The HP1, HP2 and HP3 are 3 replicates of the *H*. *meleagridis* attenuated strain (high passage, 319x).
(XLSX)

S2 Table Extended information on protein identifications. The file contains 15 folders. Folders named “Prot_HMx_all” and “Ambiguous Accessions_HMx_all” contain lists of all identified proteins and their ambiguous accessions. The total ProtScore means the score with all peptides (unique and shared). The Unused ProtScore is the score using only the unique peptides. Folders named “Prot_HMx_28_A”, “Prot_HMx_28_B”, “Prot_HMx_28_C” contain lists of proteins identified in the exoproteome of each biological replicate (A, B, C) of the low passsage *H*. *meleagridis* strain. Folders named “Prot_HMx_319_A”, “Prot_HMx_319_B”, “Prot_HMx_319_C” contain lists of proteins identified in the exoproteome of each biological replicate (A, B, C) of the high passsage *H*. *meleagridis* strain. Folders named “Ambiguous Accessions_HMx_28 or 319_A or B or C” contain lists of all ambiguous accessions for the given protein identified in the exoproteome of each biological replicate (A, B, C) of either LP (28) or HP (319) *H*. *meleagridis*. The last folder named “Contigs-accessions” contains the list of all contigs in *H*. *meleagridis* transcriptome together with their corresponding accession number and the annotation.
(XLSX)

S3 Table Protein-Peptide-Abundances and statistics. The file contains two folders. Folder named “Protein abundances incl. t-test” contains detailed output of the statistical analysis including absolute and relative abundances as well as p-values for all quantified proteins. Folder named “Peptide abundances” contains a list of unique peptides used for the quantification of each protein. Information on peptide sequence, peptide charge, mass (m/z), retention time and corresponding protein accession is given. All absolute peptide abundances are shown.
(XLSX)

## References

[1] Hess M, Liebhart D, Bilic I, Ganas P. Histomonas meleagridis-New insights into an old pathogen. Vet Parasitol. 2015;208(1–2):67–76; 10.1016/j.vetpar.2014.12.018 25576442

[2] Liebhart D, Ganas P, Sulejmanovic T, Hess M. Histomonosis in poultry: previous and current strategies for prevention and therapy. Avian Pathol. 2017;46: 1–18. 10.1080/03079457.2016.1229458 27624771

[3] Bilic I, Leberl M, Hess M. Identification and molecular characterization of numerous Histomonas meleagridis proteins using a cDNA library. Parasitology. 2009;136: 379–391. 10.1017/S0031182008005477 19154645 PMC2957082

[4] Bilic I, Jaskulska B, Souillard R, Liebhart D, Hess M. Multi-locus typing of Histomonas meleagridis isolates demonstrates the existence of two different genotypes. PLoS One. 2014;9 10.1371/journal.pone.0092438 24658534 PMC3962415

[5] Klodnicki ME, McDougald LR, Beckstead RB. A genomic analysis of Histomonas meleagridis through sequencing of a cDNA library. J Parasitol. 2013;99: 264–9. 10.1645/GE-3256.1 23075009

[6] Leberl M, Hess M, Bilic I. Histomonas meleagridis possesses three α-actinins immunogenic to its hosts. Mol Biochem Parasitol. 2010;169: 101–107. 10.1016/j.molbiopara.2009.10.007 19896981

[7] Mazet M, Diogon M, Alderete JF, Vivarès CP, Delbac F. First molecular characterisation of hydrogenosomes in the protozoan parasite Histomonas meleagridis. Int J Parasitol. 2008;38: 177–190. 10.1016/j.ijpara.2007.06.006 17697679

[8] Mazumdar R, Endler L, Monoyios A, Hess M, Bilic I. Establishment of a de novo Reference Transcriptome of Histomonas meleagridis Reveals Basic Insights About Biological Functions and Potential Pathogenic Mechanisms of the Parasite. Protist.; 2017;168: 663–685. 10.1016/j.protis.2017.09.004 29107797

[9] Monoyios A, Patzl M, Schlosser S, Hess M, Bilic I. Unravelling the differences: Comparative proteomic analysis of a clonal virulent and an attenuated Histomonas meleagridis strain. Int J Parasitol.; 2017; 10.1016/j.ijpara.2017.08.017 29203214

[10] Hess M, Kolbe T, Grabensteiner E, Prosl H. Clonal cultures of Histomonas meleagridis, Tetratrichomonas gallinarum and a Blastocystis sp established through micromanipulation. Parasitology. 2006;133: 547–554. 10.1017/S0031182006000758 16854251

[11] Hess M, Liebhart D, Grabensteiner E, Singh A. Cloned Histomonas meleagridis passaged in vitro resulted in reduced pathogenicity and is capable of protecting turkeys from histomonosis. Vaccine. 2008 10.1016/j.vaccine.2008.05.071 18586362

[12] Ganas P, Liebhart D, Glösmann M, Hess C, Hess M. Escherichia coli strongly supports the growth of Histomonas meleagridis, in a monoxenic culture, without influence on its pathogenicity. Int J Parasitol. 2012;42: 893–901. 10.1016/j.ijpara.2012.07.007 22921600

[13] Armengaud J, Christie-Oleza JA, Clair G, Malard V, Duport C. Exoproteomics: exploring the world around biological systems. Expert Rev Proteomics. 2012;9: 561–575. 10.1586/epr.12.52 23194272

[14] Ranganathan S, Garg G. Secretome: clues into pathogen infection and clinical applications. Genome Med. 2009;1: 113 10.1186/gm113 19951402 PMC2808748

[15] Roditi I. The languages of parasite communication. Mol Biochem Parasitol. 2016;208: 16–22. 10.1016/j.molbiopara.2016.05.008 27211242

[16] Gruber J, Ganas P, Hess M. Long term in vitro cultivation of Histomonas meleagridis coincides with the dominance of a very distinct phenotype of the parasite exhibiting increased tenacity and improved cell yields. Parasitology. 2017; 10.1017/S0031182017000646 28478784

[17] Erde J, Loo RRO, Loo JA. Enhanced FASP (eFASP) to Increase Proteome Coverage and Sample Recovery for Quantitative Proteomic Experiments. J Proteome Res. 2014;13: 1885–1895. 10.1021/pr4010019 24552128 PMC3993969

[18] Petersen TN, Brunak S, von Heijne G, Nielsen H. SignalP 4.0: discriminating signal peptides from transmembrane regions. Nat Methods. 2011;8: 785–786. 10.1038/nmeth.1701 21959131

[19] Bendtsen JD, Kiemer L, Fausbøll A, Brunak S. Non-classical protein secretion in bacteria. BMC Microbiol. 2005;5: 1–13. 10.1186/1471-2180-5-1 16212653 PMC1266369

[20] Shannon P, Markiel A, Ozier O, Baliga N. Cytoscape: a software environment for integrated models of biomolecular interaction networks. Genome Res. 2003; 13(11): 2498–2504; 10.1101/gr.1239303 14597658 PMC403769

[21] Chevallet M, Diemer H, Van Dorssealer A, Villiers C, Rabilloud T. Toward a better analysis of secreted proteins: The example of the myeloid cells secretome. Proteomics. 2007;7: 1757–1770. 10.1002/pmic.200601024 17464941 PMC2386146

[22] Pirkmajer S, Chibalin A V. Serum starvation: caveat emptor. AJP Cell Physiol. 2011;301: C272–C279. 10.1152/ajpcell.00091.2011 21613612

[23] Gruber J, Pletzer A, Hess M. Cholesterol supplementation improves growth rates of Histomonas meleagridis in vitro. Exp Parasitol. 2018;185:53–61. 10.1016/j.exppara.2018.01.007 29317242

[24] Klemba M, Goldberg DE. Biological Roles of Proteases in Parasitic Protozoa. Annu Rev Biochem. 2002;71: 275–305. 10.1146/annurev.biochem.71.090501.145453 12045098

[25] Piña-Vázquez C, Reyes-López M, Ortíz-Estrada G, De La Garza M, Serrano-Luna J. Host-parasite interaction: Parasite-derived and -induced proteases that degrade human extracellular matrix. J Parasitol Res. vol. 2012, Article ID 748206: 10.1155/2012/748206 22792442 PMC3390111

[26] McKerrow JH, Caffrey C, Kelly B, Loke P, Sajid M. Proteases in Parasitic Diseases. Annu Rev Pathol Mech Dis. 2006;1: 497–536. 10.1146/annurev.pathol.1.110304.100151 18039124

[27] Amin A, Nöbauer K, Patzl M, Berger E, Hess M, Bilic I. Cysteine peptidases, secreted by trichomonas gallinae, are involved in the cytopathogenic effects on a permanent chicken liver cell culture. PLoS One. 2012;7 10.1371/journal.pone.0037417 22649527 PMC3359344

[28] Hernández H, Marcet R, Sarracent J. Biological roles of cysteine proteinases in the pathogenesis of Trichomonas vaginalis. Parasite. 2014;21: 54 10.1051/parasite/2014054 25348828 PMC4209856

[29] Sajid M, McKerrow JH. Cysteine proteases of parasitic organisms. Mol Biochem Parasitol. 2002;120: 1–21. 10.1016/S0166-6851(02)00043-9 11849701

[30] Atkinson HJ, Babbitt PC, Sajid M. The global cysteine peptidase landscape in parasites. Trends Parasitol. 2009;25: 573–581. 10.1016/j.pt.2009.09.006 19854678 PMC3893884

[31] Kopitar-Jerala N. The role of cysteine proteinases and their inhibitors in the host-pathogen cross talk. Curr Protein Pept Sci. 2012;13: 767–75. 10.2174/138920312804871102 23305363 PMC3594739

[32] Schwebke JR, Burgess D. Trichomoniasis. Clinical Microbiology Reviews. 2004 pp. 794–803. 10.1128/CMR.17.4.794-803.2004 15489349 PMC523559

[33] Alvarez-Sánchez ME, Avila-González L, Becerril-García C, Fattel-Facenda L V, Ortega-López J, Arroyo R. A novel cysteine proteinase (CP65) of Trichomonas vaginalis involved in cytotoxicity. Microb Pathog. 2000;28: 193–202. 10.1006/mpat.1999.0336 10764610

[34] Cárdenas-Guerra R RE, Arroyo R, Rosa de Andrade I, Benchimol M, Ortega-López J. The iron-induced cysteine proteinase TvCP4 plays a key role in Trichomonas vaginalis haemolysis. Microbes Infect. 2013;15: 958–968. 10.1016/j.micinf.2013.09.002 24076365

[35] Hernández-Gutiérrez R, Avila-González L, Ortega-López J, Cruz-Talonia F, Gómez-Gutierrez G, Arroyo R. Trichomonas vaginalis: Characterization of a 39-kDa cysteine proteinase found in patient vaginal secretions. Exp Parasitol. 2004;107: 125–135. 10.1016/j.exppara.2004.05.004 15363938

[36] Guan Y, Zhu Q, Huang D, Zhao S, Jan Lo L, Peng J. An equation to estimate the difference between theoretically predicted and SDS PAGE-displayed molecular weights for an acidic peptide. Sci Rep. Nature Publishing Group; 2015;5: 13370 10.1038/srep13370 26311515 PMC4550835

[37] Apweiler R, Hermjakob H, Sharon N. On the frequency of protein glycosylation, as deduced from analysis of the SWISS-PROT database. Biochim Biophys Acta—Gen Subj. 1999;1473: 4–8. 10.1016/S0304-4165(99)00165-8 10580125

[38] Beynon R, Bond JS. Proteolytic Enzymes. Biochem Educ. 2000;18: 359 10.1016/0307-4412(90)90038-P

[39] Rawlings BND, Barrett AJ. Evolutionary families of metallopeptidases. Methods Enzymol. 1995;248:183–228. 10.1016/0076-6879(95)48015-3 7674922

[40] González-Miguel J, Morchón R, Siles-Lucas M, Simón F. Fibrinolysis and proliferative endarteritis: Two related processes in chronic infections? The model of the blood-borne pathogen Dirofilaria immitis. PLoS One. 2015;10: 1–22. 10.1371/journal.pone.0124445 25875022 PMC4395379

[41] Monoyios A, Hummel K, Nöbauer K, Patzl M, Schlosser S, Hess M, et al An Alliance of Gel-Based and Gel-Free Proteomic Techniques Displays Substantial Insight Into the Proteome of a Virulent and an Attenuated Histomonas meleagridis Strain. Front Cell Infect Microbiol. 2018;8 10.3389/fcimb.2018.00407 30505807 PMC6250841

[42] Cuervo P, Cupolillo E, Britto C, González LJ, e Silva-Filho FC, Lopes LC, et al Differential soluble protein expression between Trichomonas vaginalis isolates exhibiting low and high virulence phenotypes. J Proteomics. 2008;71: 109–122. 10.1016/j.jprot.2008.01.010 18541479

[43] De Jesus JB, Cuervo P, Junqueira M, Britto C, Silva-Filho FC, Sabóia-Vahia L, et al Application of two-dimensional electrophoresis and matrix-assisted laser desorption/ionization time-of-flight mass spectrometry for proteomic analysis of the sexually transmitted parasite Trichomonas vaginalis. J Mass Spectrom. 2007;42: 1463–1473. 10.1002/jms.1286 17960578

[44] EF Plow T Herren A Redlitz LM. The cell biology of the plasminogen system. FASEB J. 1995;9(10):939–45. 7615163 10.1096/fasebj.9.10.7615163

[45] Hirt RP, Noel CJ, Sicheritz-Ponten T, Tachezy J, Fiori PL. Trichomonas vaginalis surface proteins: a view from the genome. Trends Parasitol. 2007;23: 540–547. 10.1016/j.pt.2007.08.020 17962075

[46] Möllby R, Holme T, Nord CE, Smyth CJ, Wadström T. Production of phospholipase C (alpha-toxin), haemolysins and lethal toxins by Clostridium perfringens types A to D. J Gen Microbiol. 1976;96: 137–44. 10.1099/00221287-96-1-137 10344

[47] Titball RW. Bacterial phospholipases C. Microbiol Rev.;57(2):347–66. 8336671 10.1128/mr.57.2.347-366.1993 PMC372913

[48] Buist G, Steen A, Kok J, Kuipers OP. LysM, a widely distributed protein motif for binding to (peptido)glycans. Mol Microbiol. 2008;68: 838–847. 10.1111/j.1365-2958.2008.06211.x 18430080

[49] Bischofberger M, Iacovache I, Gisou Van Der Goot F. Pathogenic pore-forming proteins: Function and host response. Cell Host Microbe. 2012;12: 266–275. 10.1016/j.chom.2012.08.005 22980324

[50] Corbalan-Garcia S, Gómez-Fernández JC. Signaling through C2 domains: More than one lipid target. Biochim Biophys Acta. 2014;1838: 1536–1547. 10.1016/j.bbamem.2014.01.008 24440424

[51] Hentze MW, Castello A, Schwarzl T, Preiss T. A brave new world of RNA-binding proteins. Nat Rev Mol Cell Biol. 2018; 10.1038/nrm.2017.130 29339797

[52] Rizo J, Su TC. C2-domains, Structure and Function of a Universal Ca2+ binding Domain. J Biol Chem.1998;273(26):15879–82. 9632630 10.1074/jbc.273.26.15879

[53] Kohout SC, Corbalán-García S, Gómez-Fernández JC, Falke JJ. C2 domain of protein kinase cα: Elucidation of the membrane docking surface by site-directed fluorescence and spin labeling. Biochemistry. 2003;42: 1254–1265. 10.1021/bi026596f 12564928 PMC3666552

[54] Sheetz MP, Sable JE, Döbereiner H-G. Continuous Membrane-Cytoskeleton Adhesion Requires Continuous Accommodation To Lipid and Cytoskeleton Dynamics. Annu Rev Biophys Biomol Struct. 2006;35: 417–434. 10.1146/annurev.biophys.35.040405.102017 16689643

[55] Tsatsaronis JA, Franch-Arroyo S, Resch U, Charpentier E. Extracellular Vesicle RNA: A Universal Mediator of Microbial Communication? Trends Microbiol; 26(5):401–410. 10.1016/j.tim.2018.02.009 29548832

[56] Tkach M, Théry C. Communication by Extracellular Vesicles: Where We Are and Where We Need to Go. Cell. 2016;164: 1226–1232. 10.1016/j.cell.2016.01.043 26967288

[57] Hess M. Commensal or pathogen–a challenge to fulfil Koch’s Postulates. Br Poult Sci. Taylor & Francis; 2017;58: 1–12. 10.1080/00071668.2016.1245849 27724044 PMC5359748

[58] Ferenci T. Adaptation to life at micromolar nutrient levels: The regulation of Escherichia coli glucose transport by endoinduction and cAMP. FEMS Microbiol Rev. 1996;18: 301–317. 10.1111/j.1574-6976.1996.tb00246.x 8703508

[59] Zhao K, Liu M, Burgess RR. Adaptation in bacterial flagellar and motility systems: From regulon members to “foraging”-like behavior in E. coli. Nucleic Acids Res. 2007;35: 4441–4452. 10.1093/nar/gkm456 17576668 PMC1935009

[60] Rosales-Colunga LM, Martínez-Antonio A. Engineering Escherichia coli K12 MG1655 to use starch. Microb Cell Fact. 2014;13: 1–8. 10.1186/1475-2859-13-1 24886307 PMC4039329

[61] Flynn JM, Levchenko I, Sauer RT, Baker TA. Modulating substrate choice: The SspB adaptor delivers a regulator of the extracytoplasmic-stress response to the AAA+ protease ClpXP for degradation. Genes Dev. 2004;18: 2292–2301. 10.1101/gad.1240104 15371343 PMC517522

[62] Lee J, Hiibel SR, Reardon KF, Wood TK. Identification of stress-related proteins in Escherichia coli using the pollutant cis-dichloroethylene. J Appl Microbiol. 2010;108: 2088–2102. 10.1111/j.1365-2672.2009.04611.x 19919618

[63] Worden CR, Kovac WK, Dorn LA, Sandrin TR. Environmental pH affects transcriptional responses to cadmium toxicity in Escherichia coli K-12 (MG1655). FEMS Microbiol Lett. 2009;293: 58–64. 10.1111/j.1574-6968.2009.01508.x 19220470

[64] Mol O, Oudega B. Molecular and structural aspects of fimbriae biosynthesis and assembly in Escherichia coli. FEMS Microbiol Rev. 1996;19: 25–52. 10.1111/j.1574-6976.1996.tb00252.x 8916554

[65] Newron WA. Properties of Crystalline Tryptophanase. J Biol Chem. 1965;240:1211–8. 14284727

[66] Lee JH, Lee J. Indole as an intercellular signal in microbial communities. FEMS Microbiol Rev. 2010;34: 426–444. 10.1111/j.1574-6976.2009.00204.x 20070374

[67] Mueller RS, Beyhan S, Saini SG, Yildiz FH, Bartlett DH. Indole acts as an extracellular cue regulating gene expression in Vibrio cholerae. J Bacteriol. 2009;191: 3504–3516. 10.1128/JB.01240-08 19329638 PMC2681914

[68] Shafqat J, Höög JO, Hjelmqvist L, Oppermann UCT, Ibáñez C, Jörnvall H. An ethanol-inducible MDR ethanol dehydrogenase/acetaldehyde reductase in Escherichia coli: Structural and enzymatic relationships to the eukaryotic protein forms. Eur J Biochem. 1999;263: 305–311. 10.1046/j.1432-1327.1999.00323.x 10406936

[69] Vaňáčová Š, Rasoloson D, Rázga J, Hrdý I, Kulda J, Tachezy J. Iron-induced changes in pyruvate metabolism of Tritrichomonas foetus and involvement of iron in expression of hydrogenosomal proteins. Microbiology. 2001;147: 53–62. 10.1099/00221287-147-1-53 11160800

[70] Vizcaino JA, Csordas A, del-Toro N, Dianes JA, Griss J, Lavidas I, et al 2016 update of the PRIDE database and related tools. Nucleic Acid Res 44/D1):D447–D456. 10.1093/nar/gkv1145 26527722 PMC4702828

